# Deviating from IDSA treatment guidelines for non-purulent skin infections increases the risk of treatment failure in emergency department patients

**DOI:** 10.1017/S0950268818003291

**Published:** 2018-12-05

**Authors:** J. P. Haran, E. Wilsterman, T. Zeoli, M. Goulding, E. McLendon, M. A. Clark

**Affiliations:** 1 Department of Emergency Medicine, University of Massachusetts Medical School, Worcester, MA, USA; 2 Department of Microbiology and Physiological Systems, University of Massachusetts Medical School, Worcester, MA, USA; 3 Department of Quantitative Health Sciences, University of Massachusetts Medical School, Worcester, MA, USA

**Keywords:** Antibiotics, elderly, infectious disease, skin infections

## Abstract

The Infectious Disease Society of America (IDSA) publishes guidelines regularly for the management of skin and soft tissue infections; however, the extent to which practice patterns follow these guidelines and if this can affect treatment failure rates is unknown. We observed the treatment failure rates from a multicentre retrospective ambulatory cohort of adult emergency department patients treated for a non-purulent skin infection. We used multivariable logistic regression to examine the role of IDSA classification and whether adherence to IDSA guidelines reduced treatment failure. A total of 759 ambulatory patients were included in the cohort with 17.4% failing treatment. Among all patients, 56.0% had received treatments matched to the IDSA guidelines with 29.1% over-treated, and 14.9% under-treated based on the guidelines. After adjustment for age, gender, infection location and medical comorbidities, patients with a moderate infection type had three times increased risk of treatment failure (adjusted risk ratio (aRR) 2.98; 95% confidence interval (CI) 1.15–7.74) and two times increased risk with a severe infection type (aRR 2.27; 95% CI 1.25–4.13) compared with mild infection types. Patients who were under-treated based on IDSA guidelines were over two times more likely to fail treatment (aRR 2.65; 95% CI 1.16–6.05) while over-treatment was not associated with treatment failure. Patients ⩾70 years of age had a 56% increased risk of treatment failure (aRR 1.56; 95% CI 1.04–2.33) compared with those <70 years. Following the IDSA guidelines for non-purulent SSTIs may reduce the treatment failure rates; however, older adults still carry an increased risk of treatment failure.

## Introduction

There has been a dramatic increase in the prevalence and severity of skin and soft tissue infections (SSTIs) in the USA over the past two decades [1]. SSTIs are the second most common infection type leading to hospitalisation in the USA [1–3]. There are roughly 6.3 million physician office visits per year for SSTIs [4], and between 1993 and 2005, annual visits for SSTIs in US emergency departments (ED) increased from 1.2 to 3.4 million [5]. Non-purulent cellulitis, a common cause of SSTIs, remains poorly understood due to the lack of rapid microbiological diagnosis and remains a clinically diagnosed condition. Historically, the vast majority of cellulitis has been caused by streptococci bacterial species, often group A along with methicillin-sensitive *Staphylococcus aureus* [6, 7]. Methicillin-resistant *S. aureus* (MRSA), a common species causing purulent SSTIs, is an uncommon organism causing cellulitis [6], and *β*-lactam monotherapy remains the recommended first-line choice for non-purulent skin infections [8]. Antibiotics targeting MRSA are only needed in selective patient populations [8]. This is supported by recent randomised clinical trials of patients with cellulitis demonstrating no additional benefit of adding MRSA coverage to *β*-lactam treatment [9, 10]. Clinicians in the USA, however, prescribe antibiotic regimens that include MRSA coverage for cellulitis approximately 63% of the time in the outpatient setting [11]. Despite the multiple antibiotic classes available for the treatment of cellulitis, it has a high treatment failure rate with, on average, 20% of cellulitis patients involved in clinical trials failing treatment [12, 13].

In response to the high failure rates of cellulitis and all SSTI patients, in 2014, the Infectious Disease Society of America (IDSA) updated their guidelines for the management of SSTIs [8]. These guidelines focused on ‘promptly diagnosing SSTIs, identifying the pathogen and administering effective treatments in a timely fashion’ [8]. With an algorithm defining infection severity type and matching this to antimicrobial treatment, the guidelines simplify the management of SSTIs. The 2014 guidelines were a departure from previous versions with the creation of a treatment algorithm for three categories of SSTI severity (mild, moderate and severe) with specified treatment regimens assigned to each category. The severity categories are defined mainly by the patient's medical comorbidities (such as those with markedly impaired host defences), recent antibiotic use and severity of presenting symptoms. Despite these recommendations, clinicians typically have done a poor job in following these guidelines with adherence rates <50% [14, 15]. We have previously reported that adherence to these algorithms are poor with physicians following the guidelines for the treatment of purulent skin infections less than half of the time [14, 15]. Others have noted a similar striking lack of concordance with IDSA guidelines for both purulent and non-purulent types [16]. There is however limited literature examining real-world clinical outcomes in patients where the clinician either followed or departed from the IDSA guidelines.

The purpose of this retrospective cohort study was to assess the effectiveness of following the IDSA guidelines for the treatment of cellulitis in the outpatient setting through a descriptive analysis of the treatment and outcomes of ED patients from one large teaching and three smaller community hospitals. The objectives of this study were to: (1) describe the frequency with which treatment failure occurs among adults treated and discharged home from one of four EDs and the frequency with which clinicians practice patterns followed the severity classification treatment recommendation indicated in the 2014 IDSA guidelines for non-purulent skin infection; (2) evaluate whether not following the IDSA guidelines was an independent risk factor for treatment failure; and (3) evaluate other patient/treatment characteristics associated with treatment failure.

## Methods

### Study setting and population

We conducted a multicentre, retrospective cohort study. We identified patients 18 years and older who presented for treatment of a non-purulent skin infection between April and November in 2014 to one urban tertiary care academic centre with an annual ED census of 132 000 visits or any of three community EDs with a combined annual ED census of 100 000 visits. Patients were included in the study if they were discharged from the ED or the ED observation unit with a diagnosis of a skin or soft tissue non-purulent infection pulled from the electronic medical record discharge diagnoses listed. All patients were classified as having a suspected non-purulent infection based on discharge diagnoses. Patients already on antibiotics from a source outside of the ED were included (e.g. primary care office). Patients were excluded if they underwent an incision and drainage with expression of purulent material, if they were admitted to the hospital on their initial visit, or if they did not have follow-up visit data. Additionally, we excluded patients with a post-surgical infection, infections of the oral cavity or hidradenitis suppurativa. Patients with hidradenitis were excluded because they were not expected to improve without further surgical intervention. This study was approved by the institutional review board (IRB docket H00007714).

### Data collection

To reduce the potential for systematic error and to mitigate bias, we followed protocols for the optimal conduct of retrospective studies [17]. Prior to data abstraction activities, we *a priori* defined the pertinent predictor and outcome variables to be collected in a standardised manner. Abstractors were uniformly trained by the investigators and blinded to the study objectives and hypotheses. We utilised two different abstractors for each patient enrolled, the first one collecting data on the initial ED visit, recording demographic, historical and clinical data, while the second abstractor collected all follow-up visit data that included the principal study outcome variables. Abstractors met regularly with the investigators to review the coding rules. The investigators performed an inter-rater reliability assessment on a 10% random sample of charts.

### Variable measurement

Baseline variables were extracted by the first abstractor from the initial ED visit including socio-demographic characteristics, medical history, chief complaint and previous antibiotic use. The Charlson Comorbidity Index (CCI) was calculated and used to rank patient's medical comorbidities [18, 19]. Information pertaining to initial ED presentation, ED course and antibiotic treatments rendered was also recorded. Starting from this initial visit, all subsequent medical visits, within a 60-day window pertaining to this index infection, were reviewed by a second abstractor also using the electronic medical records. Follow-up chart review was performed by a different abstractor using electronic medical records recording any clinical visits 1 month after the initial ED visit.

### Outcomes

Our main outcome was overall treatment failure. Treatment failure was determined upon follow-up after ED discharge and defined as: any change to the initial antibiotic regimen after 24 h, including increased duration of treatment, type, dose or route of antibiotic instituted (i.e. need for intravenous antibiotics after oral treatment); admission to a hospital ward or observation unit for additional treatment; or any surgical procedures needed for treatment of the infection. The definition of treatment failure was determined *a priori* by the authors based on their previous experiences and literature on this topic [15]. If a patient met any of the above described criteria, their outcome was categorised as treatment failure, otherwise they were categorised as clinical cure. Our secondary outcome measure was admission to the hospital for treatment of the infection with a change in medical treatment. We assessed admission separately because we considered this an indication of a more severe form of failure with greater healthcare ramifications for the individual patient. All patients without documentation of treatment failure but with continued documentation or repeat visits in the medical record were classified as clinically cured.

### IDSA classification

We used IDSA guidelines to classify the severity of each patient's non-purulent skin infection upon presentation and the observed ED treatments [8]. Each case was categorised into one of three categories of non-purulent SSTIs: mild, moderate or severe as defined by the IDSA guidelines. We have previously used the IDSA guidelines in a similar retrospective chart review format [14, 15]. In brief, these guidelines define a mild infection as an uncomplicated infection without any signs of systemic infection, a moderate infection as one where the patient has signs of a systemic infection, and a severe infection when the patient has either signs of systemic inflammatory response syndrome (SIRS), an impaired host defence, associations with penetrating trauma, was an injection drug user or had evidence of MRSA infection elsewhere. Screening ambulatory patients for nasal colonisation with MRSA is not done in the ED and thus this condition could not be included in the severe category. We defined signs of systemic infection by the presence of fever either reported by the patient or recorded in the ED with a temperature >38.0 °C. We defined SIRS as the presence of one of the following: heart rate >90 beats per minute, respiratory rate >20 breaths per minute, temperature >38.0 °C, white blood count >12 000 cells/mm^3^, white blood count <4000 cells/mm^3^ or presence of >10% immature neutrophils.

The presence or absence of antibiotic classes and route of administration (intravenous or oral) determined the treatment's categorisation as mild, moderate or severe treatment class according to IDSA treatment recommendations [8]. We used the 2014 IDSA guidelines to compare the observed antibiotic treatment regimens to the expected regimens according to these guidelines. Accordingly, each patient received a score of 1 for mild, 2 for moderate and 3 for severe for their infection at the initial ED visit presentation and a second score for the treatments rendered. We then compared the clinical presentation and treatment scores with each other to determine if the observed matched the anticipated treatment class or if the patient was either over or under-treated. This formed our guideline concordant treatment index. In this index, we categorised patients into the matched group when the scores equalled each other, under-treated if the observed score was lower than the expected score and over-treated when the observed score was higher than the expected score.

### Data analysis

We used *χ*^2^ tests to compare categorical variables, and the student's *t* test for continuous variables, between patients with clinical cure and those that failed treatment. We did the same for the secondary outcome of hospital admission. Given the large sample size, data were assumed to be normally distributed. We utilised multiple-imputation which replaces missing values with multiple sets of simulated values to complete the data to address missing data in our dataset, assuming data were missing at random. Data here were missing in <5% of key variables without discernable patterns.

We used multivariable logistic regression analysis to test whether or not the IDSA severity score and guideline concordant treatment index were associated with treatment failure. The IDSA severity score and guideline concordant treatment index were the main variables of interest. To select the set of covariates for the multivariable model, we first determined important *a priori* covariates that we thought would have clinical relevance based on the literature. Covariates included patient demographics, infection characteristics and antibiotic classes of medications [14, 15]. Patient demographics included age, gender and patient comorbidities (CCI score). Age was included as a continuous variable in the initial model and also used as a categorical variable with a cutpoint of ⩾70 years of age or not determined *a priori*. We chose 70 years as an elderly age cut-off based on the changing profile of health [20] and increased mortality [21] seen starting at that age. The infection characteristics included location and the presence of proximal streaking. Any covariates with a *P* < 0.10 from unadjusted bivariate analyses were included (this resulted in facial, extremity and genital location being included in the model). The antibiotic classes with a *P* < 0.10 in the bivariate analyses were Vancomycin and third-generation Cephalosporin; however, they were not included in the multivariable model because they were both used to define the IDSA treatment categories and thus would have been collinear with this variable.

To analyse the effect of patients lost to follow-up, we performed a sensitivity analysis using best-case and worst-case scenarios where we reran the model with the patients lost to follow-up first coded as none having failed treatment and then again with them coded as all having failed treatment. We used Stata Version 13.1 (StataCorp LP, College Station, TX, USA) for all analyses.

## Results

### Characteristics of the study subjects

**Fig. 1.**
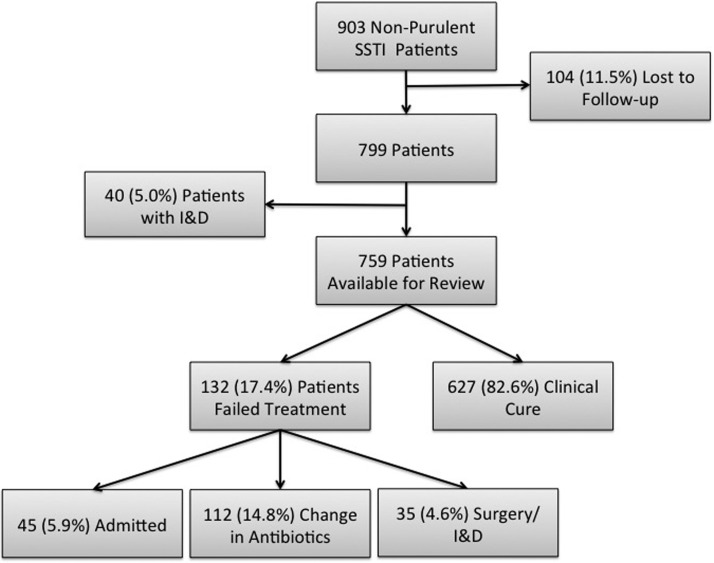
Study enrolment flow chart. SSTI, skin and soft tissue infections; I&D, incision and drainage.

During the 8-month study period in the four ED sites, there were a total of 903 patients treated and discharged home with a diagnosis of a non-purulent SSTI of which 104 (11.5%) were ultimately lost to follow-up (Fig. 1). An additional 40 patients were excluded on secondary chart review because an incision and drainage was performed, thus making the infection type purulent. Thus, out of the final cohort of 759 eligible patients, 132 (17.4%) failed treatment. We had an inter-rater reliability *κ* for all variables used in this analysis of at least 0.92.

The final study cohort had an average age of 47.8 (s.d.; 18.6) years with 13.9% being 70 years of age or older, 46.1% female and 23.6% having been treated prior to their ED visit for the same infection. Patients that failed treatment after their ED visit were older in age, with infections primarily involving an extremity not including the structures of the hand (Table 1). Women were 29% less likely than men to fail treatment (risk ratio (RR) of 0.71; 95% confidence interval (CI), 0.51–0.98) and infections of the face were 61% less likely to fail (RR 0.39; CI 0.22–0.85). We did not note any differences among patients with infections related to intravenous drug abuse, previous treatment for the same infection or currently on antibiotics.

**Table 1.** Characteristics of study patients^a^

Demographics	Clinical cure (*n* = 623)		Treatment failure (*n* = 136)		*P*
	*n* %		*n* %		
Age (s.d.)	47.1	(18.6)	50.6	(17.9)	0.034
Age ⩾70	81	(13.0)	25	(18.4)	0.07
Female	300	(48.2)	50	(37.8)	0.037
White	555	(89.1)	120	(88.2)	0.13
Hispanic	41	(6.6)	3	(2.2)	0.97
African American	21	(3.4)	8	(5.9)	0.60
Asian	10	(1.6)	1	(0.7)	0.71
Medical history					
CCI 0	451	(72.4)	94	(69.1)	0.87
CCI 1	106	(17.0)	25	(18.4)	0.57
CCI 2	50	(8.0)	12	(8.1)	0.67
CCI 3 or more	20	(3.2)	1	(0.7)	0.12
Current IVDA	24	(3.9)	6	(4.4)	0.70
Currently on Abx.	152	(24.4)	27	(19.9)	0.25
Infection characteristics					
Face	89	(14.3)	8	(5.9)	0.011
Trunk	39	(6.3)	5	(3.7)	0.28
Hand	89	(14.3)	16	(11.8)	0.53
Extremity, not hand	384	(61.6)	93	(68.4)	0.047
Buttocks	8	(1.3)	3	(2.2)	0.38
Genitals	13	(2.1)	6	(4.4)	0.09
Fever	66	(10.6)	12	(8.8)	0.43

CCI, Charlson comorbidity index; IVDA, intravenous drug abuse; Abx, antibiotics.

a Data are presented as *n* (percentages) unless otherwise indicated.

### Treatments rendered in the ED

Treatments rendered in the ED consisted of 39.1% of patients receiving intravenous antibiotics. The most common type of intravenous antibiotic used was vancomycin (glycopeptide) with 50.0% of patients receiving this class of antibiotic followed by 17.5% clindamycin (lincomycin), 10.4% third-generation cephalosporin, 8.1% first-generation cephalosporin, 6.4% aminopenicillin and 4.1% extended-spectrum penicillin. Among patients that received intravenous antibiotics in the ED (Table 2), patients treated with either a third-generation cephalosporin (RR 1.92; CI 1.13–3.29) or vancomycin (RR 1.38; CI 0.97–1.95) had an increased risk of treatment failure. There were 76 (10.0%) patients that were placed in ED observation status to receive multiple rounds of antibiotics before being discharged home. We did not detect any differences in treatment failure among the different classes of antibiotics prescribed for home (Table 2). Of note, using a single or combination of antibiotic classes covering MRSA did not change the proportion of patients that failed treatment. The most common MRSA antibiotic regimen included the combination of a first-generation cephalosporin with a sulfonamide, which occurred in 16.5% of ED patient encounters.

**Table 2.** Treatment outcome by antibiotics characteristics

Type	Clinical cure (*n* = 623)		Treatment failure (*n* = 136)		*P* value
	*n* %		*n* %		
IV ED Abx					
Vancomycin	114	(18.3)	34	(25.0)	0.08
Clindamycin	40	(6.4)	12	(8.8)	0.26
Cephalosporins 3rd	21	(3.4)	10	(7.4)	0.026
Cephalosporins 1st	20	(3.2)	4	(2.9)	0.92
Aminopenicillin	17	(2.7)	2	(1.5)	0.42
Extended-spectrum PCN	10	(1.6)	2	(1.5)	0.95
ED Observation	63	(10.1)	13	(9.6)	0.95
Home Abx					
Sulfonamide	148	(23.8)	40	(29.4)	0.11
Cephalosporins 1st	144	(23.1)	28	(20.6)	0.66
Lincomycin	116	(18.6)	24	(17.6)	0.93
Tetracyclines	42	(6.7)	9	(6.6)	0.96
Aminopenicillins	37	(5.9)	3	(2.2)	0.09
Fluroquinolones	10	(1.6)	0	(0.0)	0.14
Penicillin	8	(1.3)	1	(0.7)	0.62
Metronidazole	4	(0.6)	0	(0.0)	0.36
MRSA coverage	217	(34.8)	49	(36.0)	0.58
Cephalosporins + sulfonamide	100	(16.1)	25	(18.9)	0.40

IV, intravenous; Abx, antibiotics; ED, emergency department; PCN, penicillin; MRSA, methicillin-resistant *Staphylococcus aureus*.

Data are presented as *n* (percentages) unless otherwise indicated.

### Infection severity and treatment classifications compared with IDSA guidelines

Using the data collected from the patients at the time of ED presentation, which included medical history and symptom severity variables, we classified the IDSA SSTI severity type. The majority of patients seen and discharged home had a mild infection type (76.8%), while 9.2% had a moderate and 14.0% had a severe infection type. Using medication administration data, we classified the treatment rendered as either a mild, moderate or severe class. Patients received mild antibiotic treatment 60.8% of the time, while 19.6% received moderate and 19.5% severe antibiotic treatment class. All patients that received severe IDSA classification antibiotic treatment did so due to the administration of intravenous vancomycin. Comparing the IDSA severity class to the IDSA treatment class, we determined the proportion of patients that received treatments that matched IDSA guidelines *vs.* those that were either under or over-treated (Table 3). Guideline concordant treatment resulted in a 26% reduction in the risk of subsequent treatment failure (RR 0.74; CI 0.54–1.01) compared with not following the guidelines. Patients that were over-treated accounted for more treatment failures (Table 3).

**Table 3.** Treatment outcome by IDSA guideline comparisons

Type	Clinical cure		Treatment failure		*P* value
	*n* %		*n* %		
Mild	484	(77.7)	99	(72.8)	0.15
Moderate	54	(8.6)	16	(11.8)	0.35
Severe	85	(13.6)	21	(15.4)	0.33
Followed guidelines	361	(57.9)	64	(47.1)	0.06
Over-treated	174	(27.9)	47	(34.5)	0.07
Under-treated	92	(14.8)	21	(15.4)	0.72

Data are presented as *n* (percentages) unless otherwise indicated.

### Multivariable associations with treatment failure

In our multivariable logistic regression, which combined *a priori* clinical variables and covariates with a *P* < 0.10, patients presenting with IDSA scores of moderate and severe had more than twice the risk of treatment failure than those with a mild severity score (Table 4). When evaluating the guideline concordant treatment index, under-treating patients resulted in more than twice the risk of treatment failure while over-treatment did not increase or decrease the patient's risk after adjustment for the other covariates. The only other variable that was significantly associated with an increased risk of failure was patient age. For every decade of life, there was a 10% increase in the associated risk of treatment failure after adjusting for the other covariates which include IDSA score and under/over-treatment (Table 4). Using age as a categorical variable resulted in patients ⩾70 years being 56% more likely to fail treatment (RR 1.56; CI 1.04–2.33) than those <70 years after adjusting for the other covariates. The significance of the IDSA score, under-treatment and elderly patients having greater risk of failing treatment did not change in the sensitivity analyses.

**Table 4.** Factors significantly affecting the risk of treatment failure from multivariable logistic regression

	Risk ratio	95% CI	*P*-value
Age (10 years)	1.10	(1.01–1.20)	0.031
Above 70	1.56	(1.04–2.33)	0.029
Female	0.77	(0.54–1.04)	0.08
Previous Abx	0.74	(0.50–1.08)	0.12
CCI score	0.85	(0.69–1.04)	0.12
Facial location	0.53	(0.25–1.11)	0.09
Extremity	1.17	(0.79–1.73)	0.42
Genitals	1.72	(0.84–3.55)	0.14
Moderate symptoms	2.98	(1.15–7.74)	0.025
Severe symptoms	2.27	(1.25–4.13)	0.007
Over-treat	1.76	(0.85–3.66)	0.13
Under-treat	2.65	(1.16–6.05)	0.021

Abx, antibiotics; CCI, Charlson comorbidity index.

We ran a second logistic regression model replacing the primary outcome of treatment failure with the secondary outcome of subsequent hospital admission. In doing so, the variables that were significant in the first model remained so, and the associated risks with patient age ⩾70 years (RR 2.53; CI 1.26–5.07), severe infection type (RR 5.69; CI 2.25–14.22) and under-treatment (RR 3.82; CI 1.06–13.72), all increased considerably (Supplemental Table S1).

## Discussion

Failing initial ED outpatient treatment for a non-purulent skin infection was significantly associated with both the IDSA severity class and under-treatment according to the 2014 IDSA guideline concordant treatment index. Our findings are among the first to highlight the importance of clinicians strictly following the treatment algorithms put forth by the IDSA to reduce the risk of SSTI treatment failure. We did however find that advancing age was an independent risk factor for treatment failure. Patients aged ⩾70 years had a 56% increased risk of failing antibiotic treatment even after adjusting for the infection severity type and whether the IDSA recommended guidelines for treatment were followed. Our findings suggest that advanced age should be considered in future treatment guidelines to better reduce the risk of treatment failure in older individuals with non-purulent skin infections.

### SSTIs have high treatment failure rates

Our failure rate of 17.4% is consistent with failure rates of 15–20% using clinical trial data [9, 13, 22]. ED-based studies have found that between 18.7% and 20.5% of patients prescribed antibiotics fail treatment requiring either subsequent hospital admission, additional intravenous treatment or a change in the class of antibiotics prescribed [23, 24]. Because cellulitis consumes considerable resources to treat and fails treatment quite often, a better approach to standardise its treatment is needed.

### Clinicians have low levels of adherence to treatment guidelines

In this investigation, treatment aligned with the IDSA recommendations in just over 50% of ED patients. Experiences of clinicians having low levels of adherence to published guidelines are not unique to the USA or SSTIs. In Europe, the Clinical Resource Efficiency Support Team (CREST) publishes guidelines similar to the IDSA for treatment of cellulitis, but when studied in practice clinicians still over-treat 43% of the time [25, 26]. In the USA, one in three antibiotics prescribed in the ambulatory setting are inappropriate [27]. The Centers for Disease Control and Prevention predict that at least 50% of antibiotics prescribed for acute respiratory conditions are unnecessary [28], while it has been shown that only 52% of antibiotic prescriptions given for sinus infections, middle ear infections and pharyngitis match recommendations based on established guidelines [29]. This inappropriate and excessive treatment with broad-spectrum antibiotics leads to antimicrobial resistance and has never been shown to improve clinical outcomes. We have shown here that patients that were over-treated did not have a reduction in failure risk; however, those that were under-treated did have an increased risk. This demonstrates how following the IDSA guidelines leads to fewer treatment failures and can reduce unnecessarily broad antibiotic use. A next logical approach would be to prospectively apply these guidelines in the clinical setting to determine if changing practice to better align with the IDSA guidelines results in a reduction in treatment failures.

### The elderly have an increased risk of treatment failure

Advancing age is an important clinical factor to take into account when treating patients for bacterial infections and has been included in other treatment algorithms such as those for the treatment of community-acquired pneumonia [30, 31]. Patient's age is not included in the IDSA SSTI algorithms. Here, we found that elderly patients treated and discharged home had a 56% increased risk of treatment failure and almost three times the risk of admission after adjusting for adherence to the IDSA guidelines. Age is an important variable to consider given that elderly patients’ immune system defence mechanisms weaken over time making them more susceptible to many types of bacterial infections when compared with younger adults [32]. Combining this with the fact that SSTIs are common in the elderly [33] makes defining differential treatment decisions by age essential to reducing failure rates. The way in which clinicians should treat elders with a non-purulent infection differently from younger patients (i.e. antibiotic class or route choice, admission) still needs defining, and presents an important area for future work.

### Study strengths and limitations

Our study has some limitations. First, this is a retrospective cohort study design, which may introduce selection and information biases. However, results from sensitivity analyses suggest that selection bias from loss to follow-up did not influence the main effects of IDSA treatment and age on the outcome of treatment failure. We also used discharge diagnosis rather than ICD codes which may have missed some patients that would have been included using ICD codes. However, we attempted to limit this by looking at all possible relevant discharge diagnoses (i.e. sepsis) to ensure we did not miss these patient types. Second, clinicians have had difficulty in appropriately diagnosing cellulitis [34], which may have led to misclassification of the eligibility criteria. We did not attempt to adjust for this type of misclassification in order to observe how the guidelines perform in real-life clinical practice. Another limitation is the way in which we defined signs of systemic infection, that being the presence of a fever. It is possible that in practice clinicians may use other data sources than fever alone to make treatment determinations aligning with the IDSA moderate infection type, which are just not available through review of the medical record. Another limitation is that follow-up was done through chart review that did not include facilities outside of our medical record. We attempted to limit this by reviewing primary care notes for all patients to probe to see if the patient was seen at another facility. Additionally, we excluded patients if they did not have follow-up visit data. Finally, the majority of patients whose ED treatment was classified under the severe category were done so due to the inclusion of intravenous vancomycin into their treatment regimen. In the IDSA guidelines, vancomycin plus another class of antibiotics places the treatment under the severe category unless culture sensitivities are available, which is often not the case in the ED. It may be that either one of two doses of vancomycin are inappropriate treatment before discharge; however, we did not detect this association in our bivariate analysis nor is this stipulated in the guidelines. This study's balancing strengths are that it is the first to examine concordance with IDSA SSTI guidelines in relation to patient outcomes in practice in a large cohort of patients treated from multiple sites that include both academic and community settings.

## Conclusions

In summary, our findings suggest that adherence to the IDSA guidelines for non-purulent SSTIs can reduce the treatment failure rate among ambulatory patients, thus improving their care and reducing unnecessary antibiotic use. The next step is to prospectively apply these guidelines and observe the treatment failure rates pre- and post-intervention. By educating and developing simple aids to guide clinicians, we might start to improve both adherence rates and patient outcomes. Further, our study suggests that advancing age is an important clinical factor to include in future SSTI treatment guidelines given the increased prevalence and treatment failure rates among this vulnerable group of patients. Adjusting antimicrobial treatment among the elderly may be an important avenue to further reduce the treatment failure rates that have historically been associated with non-purulent SSTI outcomes.

## Supplementary material

For supplementary material accompanying this paper visit https://doi.org/10.1017/S0950268818003291. click here to view supplementary material
