# Supplementary material for: Deviating from IDSA treatment guidelines for non-purulent skin infections increases the risk of treatment failure in emergency department patients
Source: Epidemiol Infect. 2018 Dec 5;147:e68. doi: 10.1017/S0950268818003291 (PMC6518578; doi:10.1017/S0950268818003291)
Supplement: Supplementary file 1 [file S0950268818003291sup001.docx]

**Supplemental Table 1.** Factors significantly affecting the risk of treatment failure from multivariable logistic regression with secondary outcome

|  |  | |  | |
| --- | --- | --- | --- | --- |
|  | Risk Ratio | 95% CI | p-value |  |
| Above 70 | 2.73 | (1.23-1.75) | 0.005 |  |
| Female | 0.47 | (0.24-0.91) | 0.026 |  |
| IDSA Score 2 | 3.08 | (0.42-22.34) | 0.27 |  |
| IDSA Score 3 | 7.04 | (2.38-20.86) | <0.001 |  |
| Over Treat | 2.14 | (0.60-7.56) | 0.26 |  |
| Under Treat | 4.32 | (1.00-18.86) | 0.05 |  |
| After adjusting for gender**,** previous antibiotic exposure, Charlson comorbidity index, and location of infection (face, extremity and genitals). | | | | |
